# Porcine deltacoronavirus causes diarrhea in various ages of field-infected pigs in China

**DOI:** 10.1042/BSR20190676

**Published:** 2019-09-16

**Authors:** Bingxiao Li, Lanlan Zheng, Haiyan Li, Qingwen Ding, Yabin Wang, Zhanyong Wei

**Affiliations:** 1 The College of Animal Science and Veterinary Medicine, Henan Agricultural University, Zhengzhou, Henan 450002, P. R. China; 2 Key Laboratory for Animal-derived Food Safety of Henan Province, Zhengzhou, Henan 450002, P. R. China

**Keywords:** Diarrhea, Histopathology, PDCoV, Pig age, qRT-PCR

## Abstract

Porcine deltacoronavirus (PDCoV) is a novel coronavirus that causes acute diarrhea in suckling piglets. In Henan province of China, three swine farms broke out diarrhea in different ages of pigs during June of 2017, March of 2018 and January of 2019, respectively. PCR method, Taqman real-time RT-PCR method, sequencing, histopathology and immunohistochemistry (IHC) were conducted with the collected samples, and the results showed that PDCoV was detected among the suckling piglets, commercial fattening pigs and sows with diarrhea. PDCoV-infected suckling piglets were characterized with thin and transparent intestinal walls from colon to caecum, spot hemorrhage at mesentery and intestinal bleeding. PDCoV RNA was detected in multiple organs and tissues by Taqman real-time RT-PCR, which had high copies in ileum, inguinal lymph node, rectum and spleen. PDCoV antigen was detected in the basal layer of jejunum and ileum by IHC. In this research, we found that PDCoV could infect various ages of farmed pigs with watery diarrhea and anorexia in different seasons in a year.

## Introduction

PDCoV is an enveloped, positive-sense, single-stranded RNA virus that belongs to the subfamily *Coronavirinae* in the family *Coronaviridae* within the order *Nidovirales* [1]. This novel virus was initially reported in Hong Kong in 2012 [2], and then outbreak of PDCoV in pig herds was announced in the United States in early 2014 [3,4]. Since then, the detection of PDCoV was reported subsequently in many countries, such as South Korea, Canada, China, Vietnam and Japan [5–9]. PDCoV could cause acute diarrhea, vomiting, dehydration and even lead to death in nursing piglets, with the main lesion of villous atrophy in intestines [10–13]. The prevalence of PDCoV in Henan province of China was about 23.49%, and up to 36.43% in suckling piglets [14,15]. Infected sows usually did not show obviously clinical signs so that the PDCoV detection in sows was often ignored.

Besides PDCoV, there are several main viral pathogens, which cause porcine diarrhea that endanger the healthy development of swine industry. Transmissible gastroenteritis virus (TGEV), the re-emerged porcine epidemic diarrhea virus (PEDV), and the novel swine acute diarrhoea syndrome coronavirus (SADS-CoV), which all belong to genus *Alphacoronavirus* [16], have similar clinical symptoms with watery diarrhea, vomiting and dehydration, and similar pathological features with small intestinal enterocyte necrosis and villous atrophy in neonatal piglets. The co-infection of PDCoV with these viruses is common in clinic. However, PEDV could cause severe diarrhea and high mortality (up to 100%) in piglets worldwide [17]. The prevalence of PEDV infection was higher in cold season, especially in January and February, compared with that in warm seasons [18,19]. With TGEV infection, the mortality rate of neonatal piglets comes up to 100%, especially in piglets no more than 2 weeks of age [20,21]. SADS-CoV mainly infected newborn pigs which are less than 5 days of age, and the mortality rate was 90% [16].

During June of 2017, March of 2018 and January of 2019, three swine farms in different cities (Zhumadian, Zhoukou, Nanyang) of Henan Province, China, broke out diarrhea diseases in different ages of pigs with high mortality in suckling piglets. The diarrhea disease in the three farms all first broke out at sows with vomiting and mild diarrhea, and then the newborn piglets developed acute, watery diarrhea, anorexia, rough hair and vigorous prostration with high mortality rate about 60%. Fattening pigs developed diarrhea with growth retardation and anorexia. However, some sows with vomiting and diarrhea recovered 1 day later, which showed transient diarrhea.

In the present study, the fecal samples of pigs with different ages were collected and identified by RT-PCR of viruses which cause diarrhea. After the pathogen causing diarrhea in the three swine farms was determined, virus distribution in tissues of the infected piglets was assessed by Taqman real-time RT-PCR, and the histopathological changes and antigen were observed by Hematoxylin and Eosin (H.E) staining and IHC.

## Materials and methods

### Clinical sample collection

**Figure 1:**
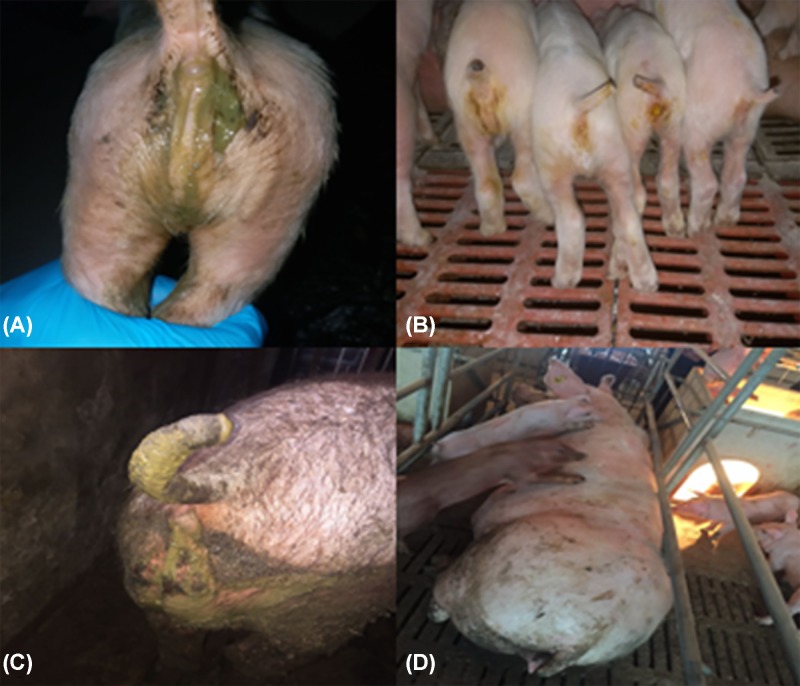
Clinlcal symptoms Clinical assessment of PDCoV-infected pigs with acute, severe watery diarrhea, depression and lethargy. Abundant like gray cement, watery stools were also observed around the perianal region of fattening pigs and sows. (**A,B**) 7-day-old pigs; (**C**) 5-month-old fatting pig; (**D**) 2-year-old sow.

From June of 2017 to January of 2019, the Key Laboratory for Animal-derived Food Safety in Henan Agricultural University received clinical samples from three swine farms that suffered from diarrhea disease among the farms, with high mortality rate in suckling piglets. Farm A was a 300-sow breed-to-finisher farm in Zhumadian City of Henan Province, farm B was a 300-sow breed-to-finisher farm in Zhoukou City of Henan Province, and farm C was a 150-sow breed-to-finisher farm in Nanyang City of Henan Province. In the three swine farms, watery diarrhea and vomit was first found in sows, and by the following day the newborn piglets showed acute, watery diarrhea with high mortality rate, and then this disease spread to all pigs in the farms (Figure 1).

55 samples (including 8 suckling piglets, 8 fecal samples of suckling piglets, 10 fecal samples of weaned pigs, 13 fecal samples of fattening pigs and 16 fecal samples of sows) were collected from farm A. 55 samples (including 6 suckling piglets, 10 fecal samples of suckling piglets, 12 fecal samples of weaned pigs, 12 fecal samples of fattening pigs and 15 fecal samples of sows) were collected from farm B. 67 samples (including 6 suckling piglets, 15 fecal samples of suckling piglets, 13 fecal samples of weaned pigs, 17 fecal samples of fattening pigs and 16 fecal samples of sows) were collected from farm C. Moreover, three suckling piglets from each swine farm were chosen to necropsy. The intestinal sections, small intestinal content (SIC), tissues of heart, liver, spleen, lung, kidney, intestines, inguinal lymph node and serum were collected during the suckling piglets necropsy.

### Viral RNA extraction

All the collected fecal samples and intestinal contents were diluted fivefold with phosphate-buffered saline (PBS) (Boster, China). About 0.1 g tissues of heart, liver, spleen, lung, kidney, intestines and inguinal lymph node were collected, grinded and diluted fivefold with PBS. The samples were centrifuged at 1847 ***g*** at 4°C for 20 min. The supernatants were collected for viral RNA extraction. Viral RNA was extracted using the TRIzol Reagent (Invitrogen, Carlsbad, CA, U.S.A.) according to the manufacturer’s instructions. The RNA concentration was determined by measuring absorbance at 260 nm (*A*_260_) using Nanodrop (Thermo Fisher Scientific, U.S.A.).

### RT-PCR detection

RNA was used as a template to generate cDNA using Prime Script RT Reagent Kit (Takara, Biotechnology, China). Then PDCoV, PEDV, TGEV, SADS-CoV and porcine rotavirus (PoRV) were detected by RT-PCR. Primers of PDCoV, PEDV, TGEV and PoRVA/B/C were designed and preserved by the Key Laboratory for Animal-derived Food Safety of Henan Province. Primers of SADS-CoV were synthetized that targeted the mostly conserved gene of SADS-CoV [22]. The primers were shown in Table 1.

**Table 1:** Primers used for amplification of viruses

Primer identification	Sequence (5′–3′)	Fragment (bp)	Tm (°C)
PDCoV	F:GACCCTAAATCTGCCGTTAGAG	547	53
	R:TGTTGGAGAGGTGAATGCTATG		
PEDV	F:GCATTTCTACTACCTCGGAA	750	58
	R:GCGATCTGAGCATAGCCTGA		
TGEV	F:CGCTATCGCATGGTGAAG	324	58
	R:GGATTGTTGCCTGCCTCT		
SADS-CoV	F:ATGACTGATTCTAACAACAC	686	60
	R:TTAGACTAAATGCAGCAATC		
PoRV-A	F: ACCATCTACACATGACCCTC	171	54
	R: GGTCACATAACGCCCC		
PoRV-B	F:AATTGGGGHAATGTGTG	102	50
	R:TCGCCTAGTCYTCTTTATG		
PoRV-C	F:ACAGTATTTCAGCCAGGDTTTC	237	54
	R: AGCCACATAGTTCACATTTCATC		

### Genomic analysis

After RT-PCR detection, we chose one positive sample in each farm randomly, and the S gene was amplified. Specific primers of PDCoV S gene were designed (F:5′-CAGGACGCCTTCTTGTGA-3′, R:5′-GGGTTCGGCTTGGAGTAG-3′) to amplify the 3692 bp of S gene on the conditions of 95°C for 3 min, followed by 35 cycles of 95°C for 15 s, 58°C for 15 s, 72°C for 4 min and finally 72°C for 5 min. The sequenced S genes were assembled with DNAStar Lasergene 7.0, and then used in sequence alignment and phylogenetic analyses using the neighbor-joining method in MEGA 6.0 software (http://www.megasoftware.net/).

### Analysis the PDCoV viral RNA distribution by TaqMan real-time RT-PCR

Based on the M gene sequence of PDCoV in GenBank, a pair of primers was designed. The forward primer was 5′-CTATGTCTGACGCAGAAGAGTG-3′ and the reverse primer was 5′-GATGTGCCGCTTATTGCA-3′. Then it was cloned into pMD18-T vector to generate the recombinant plasmid. Another pair of primers and TaqMan probe were designed based on the M gene sequence to develop a TaqMan qRT-PCR method. The forward primer was 5′-GACTCCTTGCAGGGATTATGG-3′ and the reverse primer was 5′-GCTTAACGACTGGTGTGAGAA-3′. The probe was 5′-FAM-ATGGGTACATGGAGGTGCATTCCC-TAMRA-3′. The TaqMan real-time RT-PCR reaction system was 12.5 μL of Ex Taq premix (Probe qPCR) (Takara, Biotechnology, China), 0.5 μL (25 mol/μL) of forward and reverse primers, 1 μL probe, 2 μL of PDCoV cDNA, and H_2_O was added up to 25 μL. RT-PCR amplification program was pre-incubated at 95°C for 30 s; 40 cycles at 95°C for 5 s, 60°C for 30 s. The detection limit of TaqMan real-time RT-PCR was 3.7 log_10_ GE/mL for the original fecal sample and intestinal contents, 3 log_10_ GE/mL for the serum sample.

### Gross pathology and histopathology

During necropsy, the small intestines (duodenum and ileum) and large intestines (cecum and colon) and other major organs, including lung, heart, kidney and spleen were examined grossly. Samples collected from these tissues were fixed by 10% neutral buffered formalin for 48 h and for histopathological examination as described previously [23]. Fixed tissues were embedded, sectioned, and stained with Mayer’s H.E for light microscopy examination. The length of ten villi and crypts of jejunum was measured and the mean of jejunum villous height: crypt depth (VH: CD) ratios was calculated as described [23].

### IHC for the detection of PDCoV antigen

Jejunum and ileum are the primary infection sites of PDCoV, and PDCoV antigen is observed both in the small intestines and large intestines [24]. So we chose small and large intestines for the detection of PDCoV antigen by IHC. The prepared tissue samples were formalin-fixed, and paraffin-embedded tissue sections were de-waxed in xylene and rehydrated in decreasing 95, 85, 75% concentrations of ethanol for 1 min. Antigen retrieval was performed in citrate buffer (pH 6.0) at 95°C for 20 min. Slides were blocked with 5% bovine serum albumin (BSA) (Boster, China) at 37°C for 1 h, and then incubated with rabbit anti-PDCoV-N protein polyclonal antibody overnight at 4°C in a humidified chamber. Stained sections were then incubated with biotinylated secondary antibodies (Boster, China) at 37°C in a humidified chamber for 1 h, and treated with strept avidin–biotin complex (SABC) (Boster, China) for 1 h. Slices were washed three times with PBS after each incubation step, and positive cells were visualized with the treatment of diaminobenzidine (DAB) [25]. Sections were counterstained with Hematoxylin and images were obtained using a light microscope.

## Results

### The main diarrhea-relating pathogens detection results

The collected samples were detected for PDCoV, PEDV, TGEV, SADS-CoV and PoRVA/B/C by RT-PCR. The results showed that in farm A, eight SIC samples from eight suckling piglets were positive for PDCoV, and 39/47 fecal samples were positive for PDCoV which included 8/8 fecal samples of suckling piglets, 8/10 fecal samples of weaned pigs, 10/13 fecal samples of fattening pigs, and 13/16 fecal samples of sows. In farm B, five SIC samples of six suckling piglets were positive for PDCoV, and 29/49 fecal samples were positive for PDCoV which included 8/10 fecal samples of suckling piglets, 6/12 fecal samples of weaned pigs, 6/12 fecal samples of fattening pigs and 9/15 fecal samples of sows. In farm C, six SIC samples of six suckling piglets were positive for PDCoV, and 36/61 fecal samples were positive for PDCoV which included 12/15 fecal samples of suckling piglets, 6/13 fecal samples of weaned pigs, 8/17 fecal samples of fattening pigs and 10/16 fecal samples of sows (Table 2). We chose one positive sample in each farm for sequencing, and the three samples were identified as PDCoV.

**Table 2:** PDCoV detection results by RT-PCR

	fecal samples				SIC of suckling piglets
	suckling piglets	weaned pigs	fattening pigs	sows	
Farm A	8*/8	8*/10	10*/13	13*/16	8*/8
Farm B	8*/10	6*/12	6*/12	9*/15	5*/6
Farm C	12*/15	6*/13	8*/17	10*/16	6*/6
Total	28*/33	20*/35	24*/42	32*/47	19*/20

* positive number of PDCoV.

The prevalence of PDCoV in suckling piglets of the three farms was up to 84.8%, and 68.1% in sows. There was the same prevalence rate (57.1%) in weaned pigs (30–60 days old) and fattening pigs (over 90 days old) (Table 2). All the infected pigs had vomit and diarrhea symptoms, but some sows infected with PDCoV showed transient diarrhea only lasting for 1 day. In addition, RT-PCR results of PEDV, TGEV, SADS-CoV and PoRVA/B/C detection were all negative.

### Characterization of the PDCoV epidemic strains

**Figure 2:**
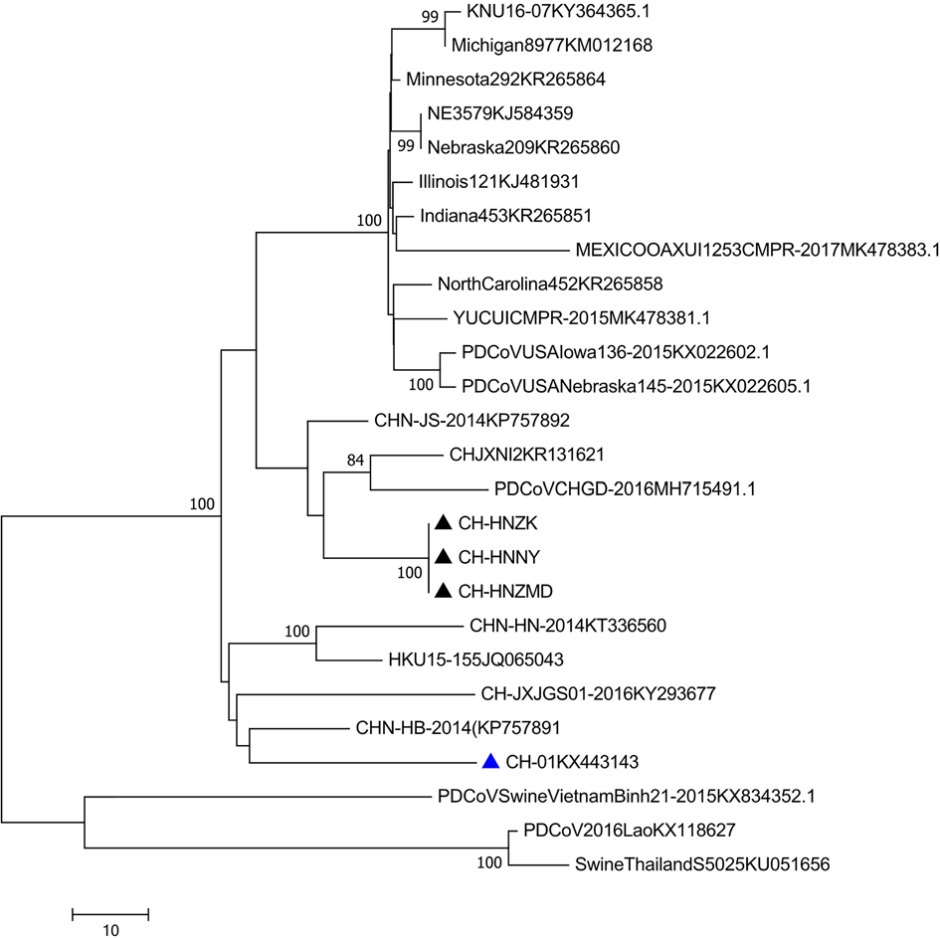
Phylogenetic analysis of the S genes from different PDCoV strains The phylogenetic tree was constructed and analyzed using the neighbor-joining method of MEGA 6.0 software (http://www.megasoftware.net). Bootstrap values were calculated with 1000 replicates. Reference sequences obtained from GenBank are indicated by strain names and GenBank accession numbers. The S genes of PDCoV isolated from three swine farms in the present study are indicated with black triangles. The first strain of PDCoV S gene isolated by our lab is indicated with a blue triangle.

The PDCoV S genes amplified from the three farms were sequenced (CH-HNZK, CH-HNNY, CH-HNZMD) and phylogenetic tree was constructed using the three sequenced S genes and other PDCoV S genes obtained from NCBI (Figure 2). It showed that the three strains of PDCoV clustered in same group, and had close relationship with other PDCoV strains isolated in China, which indicated that the PDCoV prevalence in Henan province was consistently with other PDCoV strains in China.

### Pathological lesion of PDCoV-infected piglets

**Figure 3:**
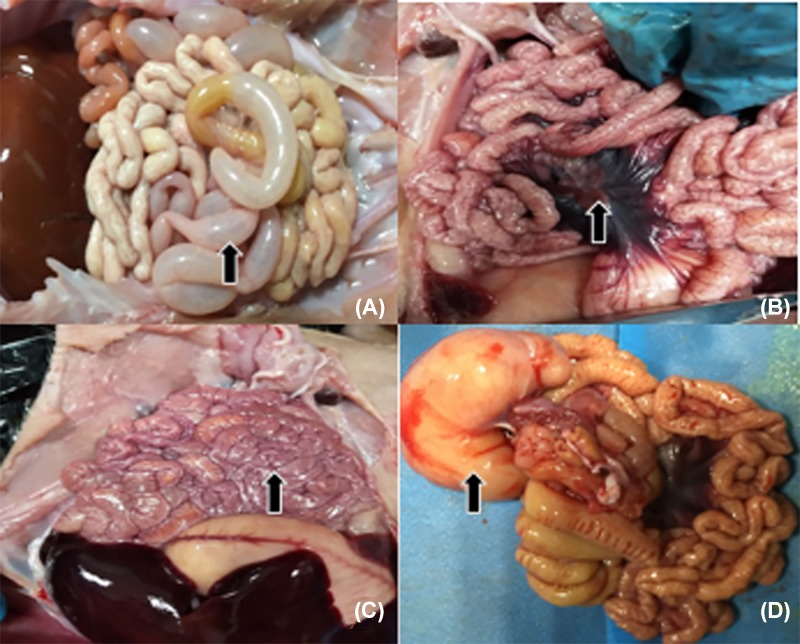
Intestinal changes in PDCoV infected piglets (**A**) Piglets showed thin and transparent intestinal walls from colon to caecum (arrows). (**B**) Mesentery with spot hemorrhage (arrows). (**C**) Intestinal bleeding (arrows). (**D**) Stomach filled with curdled milk and accumulation of large amounts of yellow fluid in the jejunum lumen (arrows).

Nine piglets (three piglets were chosen in each farm) that positive for PDCoV were euthanized for macroscopic examination. The results showed that all infected piglets characterized by thin and transparent intestinal walls from colon to caecum (Figure 3, panel A) and spot hemorrhage at mesentery (Figure 3, panel B). We also found intestinal bleeding (Figure 3, panel C) and the stomach was filled with curdled milk and accumulation of large amounts of yellow fluid in the jejunum lumen (Figure 3, panel D).

### Virus distribution in the PDCoV field-infected piglets

**Figure 4:**
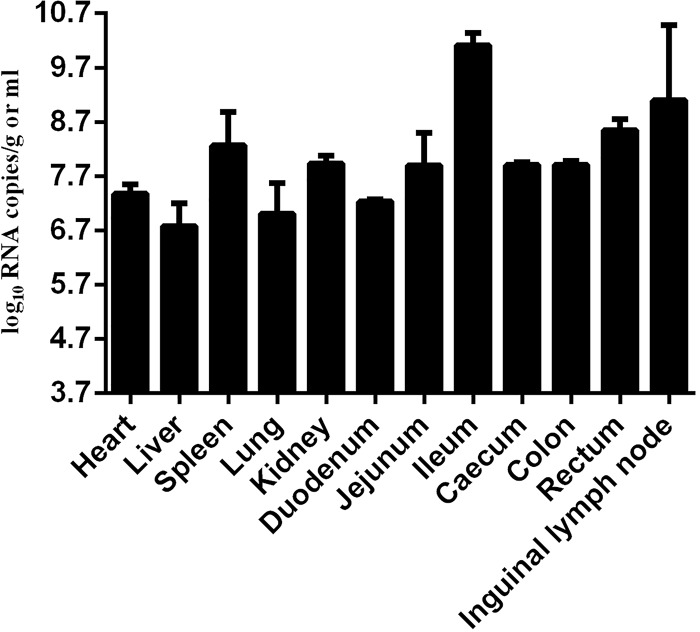
PDCoV distribution in various tissues The virus copies (log_10_ GE/µg of total RNA) were mean virus copy of nine piglets. High PDCoV RNA copies were detected in ileum, inguinal lymph node, rectum and spleen. The highest PDCoV RNA copy was detected in ileum. Standard error bars are shown in each tissue.

PDCoV distribution in different tissues of the piglets was examined by Taqman real-time RT-PCR. PDCoV RNA distributed systemically with various copies among tissues, and high PDCoV RNA copies were detected in ileum, inguinal lymph node, rectum and spleen (Figure 4). The highest PDCoV RNA copy was detected in ileum (10.0 ± 0.22 log_10_ GE/µg of total RNA). And the PDCoV RNA copy was 8.6 ± 0.18 log_10_ GE/µg in serum.

### Histopathology and immunohistochemistry on the intestinal lesions of the PDCoV field-infected piglets

**Figure 5:**
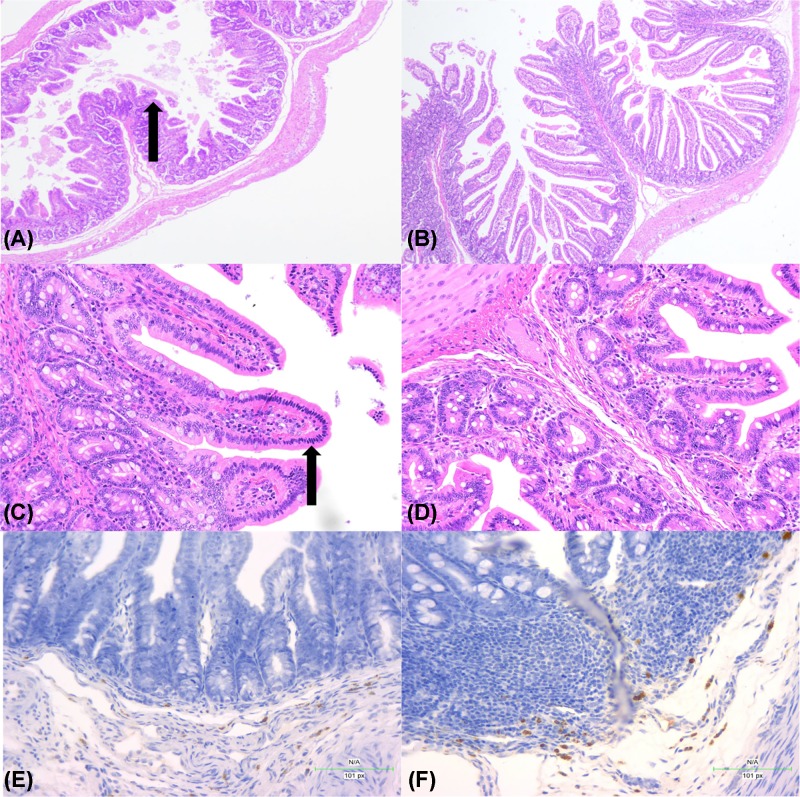
Microscopic lesions and IHC staining (**A**) H.E-stained jejunum of PDCoV-infected piglet with intestinal villus atrophy and acute diffuse jejunitis (original magnification ×40) (arrows). (**B**) H.E-stained jejunum tissue section of a control pig. (**C**) H.E-stained ileum of PDCoV infected piglet with intestinal acute, jejunitis diffuse cell proliferation and ileitis. (original magnification ×100). (arrows). (**D**) H.E-stained ileum tissue section of a control pig. (**E**) Section of jejunum of PDCoV-infected piglet, showing basal layer of intestine are positive for PDCoV RNA (original magnification ×400). (**F**) Section of ileum of PDCoV-infected piglet, with basal layer of intestine are positive for PDCoV RNA (original magnification ×400).

Intestinal tracts of PDCoV positive piglets were investigated after H.E staining, and some obvious pathological changes were found. Sections of middle jejunum to caecum showed diffuse intestinal villus blunting, fusion and enterocyte attenuation (Figure 5). No lesions were seen in other organs. The mean VH: CD was 2.33 ± 0.58 in duodenum, 1.71 ± 0.81 in jejunum, 1.88 ± 0.74 in ileum and 3.02 ± 0.11 in cecum, respectively.

PDCoV antigen was detected in the cytoplasm of villous enterocytes in jejunum and ileum (Figure 5E,F). Duodenum and cecum also showed PDCoV positive by IHC staining slightly. PDCoV was not observed in other examined sections of intestine.

## Discussion

PDCoV has been detected in many countries, and previous researches showed that the prevalence of PDCoV was mainly focus on suckling piglets with the mortality rate from 40 to 80% [14,15]. PDCoV was reported in Ohio of U.S.A. in February 2014 that with diarrhea in sows and piglets [4]. Another PDCoV infection was reported in Thailand, with acute diarrhea in piglets, gilts and sows [26]. In our study, PDCoV positive infection was not only found in suckling piglets and weaned pigs, but also detected in commercial fattening pigs and sows. Especially, pigs of different ages with PDCoV infection showed clinical symptoms such as watery diarrhea, anorexia and wasting, indicated that the prevalent surveillance of PDCoV should cover pigs of different ages in clinic.

Under our investigation in the three swine farms, we found that PDCoV was the main pathogen of diarrhea in these swine farms. Among 177 samples we collected, 123 samples were positive of PDCoV, with 69.5% positive rate, which meant that the diarrhea in the three swine farms was mainly caused by PDCoV. In addition, among the 47 fecal samples of sows, there were 32 samples positive with PDCoV, which suggested that PDCoV could lead to diarrhea in sows independently. PDCoV is often co-infected with PEDV and/or TGEV, which bring huge economic loss to swine farms [27–29], while in the present study, we found that PDCoV monoinfection could cause diarrhea disease in pigs of different ages. And the mortality rate of suckling piglets is higher than that of other ages of pigs, which had the same results with the previous research that PDCoV mainly focus on suckling piglets and cause severe mortality rate [14,15].

Previous reports showed that PDCoV was observed mainly in the small and large intestines, like the PEDV and TGEV infection, and could be detected in multiple organs such as heart, liver, spleen, lung, kidney and stomach in the PDCoV experimental-infected pigs [10]. In this research, PDCoV viral RNA was also detected in intestines, heart, spleen, lung, kidney and many other organs by Taqman real-time RT-PCR [30,31]. This result showed that there was the similarity in viral distribution in the tissues and organs between field and experimental PDCoV-infected pigs. The number of viral RNA copy in intestinal tract was higher than that in other tissues. It is known that PDCoV antigen captured mainly in villous enterocytes of the small and large intestines [30,31], but we detected some PDCoV antigen-positive cells in the intestinal crypts, which had the same result with Jung’s report [32].

PDCoV outbroke in the three different farms in current study in January, March and June, respectively, indicating that PDCoV was highly pathogenic not only in cold months, but also in warmer months. PDCoV was first reported in early February of 2014 in the United States, in March of 2014 in Canada, in April of 2014 in Korea [4–6]. It seemed that like PEDV and TGEV [21,22], disease caused by PDCoV infection mainly peaks in colder months between January and April. However, in the present study, one swine farm outbroke PDCoV in June, which is a very hot month in Henan Province of China, indicating that we need to continue monitoring the prevalence of PDCoV in all the seasons.

In conclusion, we found that field infection of PDCoV can lead to diarrhea, wasting and other clinical symptoms not only in sucking piglets and weaned pigs, but also in fattening pigs and sows in both cold and warm months, which indicated that PDCoV could infect various ages of farmed pigs with watery diarrhea.

## Abbreviations

H.E: Hematoxylin and Eosin
IHC: immunohistochemistry
PDCoV: porcine deltacoronavirus
PEDV: porcine epidemic diarrhea virus
PoRV: porcine rotavirus
SADS-CoV: swine acute diarrhoea syndrome coronavirus
SIC: small intestinal content
TGEV: transmissible gastroenteritis virus
VH: CD: villous height: crypt depth
